# Complete chloroplast genome data of *Ranunculus membranaceus* (Ranunculaceae), an important medicinal plant species

**DOI:** 10.1016/j.dib.2023.109416

**Published:** 2023-07-16

**Authors:** Yanmei Ren, Mengyan Wang, Zuoyi Wang, Lin Wu, Ji-Zhong Wan, Huakun Zhou, Zhen Ma, Chunhui Zhang

**Affiliations:** a State Key Laboratory of Plateau Ecology and Agriculture, College of Eco-Environmental Engineering, Qinghai University, Xining, Qinghai 810016, China; b Qinghai Provincial Key Laboratory of Restoration Ecology for Cold Regions, Northwest Institute of Plateau Biology, Chinese Academy of Sciences, Xining, Qinghai 810008, China; c Key Laboratory of Adaptation and Evolution of Plateau Biota, Northwest Institute of Plateau Biology, Chinese Academy of Sciences, Xining, Qinghai 810008, China; d Qinghai Haibei National Field Research Station of Alpine Grassland Ecosystem, Northwest Institute of Plateau Biology, Chinese Academy of Sciences, Xining, Qinghai 810008, China

**Keywords:** Complete chloroplast, Genome, Phylogeny, Ranunculaceae, *Ranunculus*

## Abstract

The perennial alpine herb *Ranunculus membranaceus* (Ranunculaceae) has significant medicinal value. The complete chloroplast genome of *R. membranaceus* was sequenced by high-throughput Illumina sequencing Platform Illumina NovaSeq 6000. The circular genome is 156,028 bp in size, including two inverted repeats (IRs) of 25,361 bp, a large single-copy (LSC) region of 85,491 bp, and a small single-copy (SSC) region of 19,815 bp. A total of 128 genes were annotated, namely 84 protein-coding genes (PCGs), 36 tRNA genes, and eight rRNA genes. Two phylogenetic trees of 18 species of the tribe Ranunculeae species were constructed with *Meconopsis punicea* as the outgroup based on the whole chloroplast genomes and the concatenated sequence of PCGs, respectively. Phylogeny showed that *R. membranaceus* was closely related to *R. yunnanensis*. These data enrich knowledge of Ranunculaceae genetics and will contribute to further studies of *R. membranaceus* in molecular breeding, genetic transformation, species identification, genetic engineering and phylogenetic research.

**Specifications Table**

**Table:** 

Subject	Biological Science
Specific subject area	Omics: Genomics
Type of data	Table Figure
How the data were acquired	The chloroplast genomes of *Ranunculus membranaceus* were sequenced using an Illumina Novaseq 6000 platform.
Data format	Raw Analyzed
Description of data collection	The chloroplast DNA was sequenced using an Illumina NovaSeq 6000 platform. The SPAdes (version 3.14.0) and Flye (version 2.8.3, online version: https://github.com/fenderglass/Flye) were used to assemble the chloroplast genome of both taxa. The reference genome and splicing results were compared using BLAST version 2.2.30^+^ with two chloroplast genomes as references and annotated using GeSeq (online version: https://chlorobox.mpimpgolm.mpg.de/geseq.html) with manual correction. The circular chloroplast genome map was drawn using OGDRAW.
Data source location	City: Menyuan City, Qinghai Province. Country: China. Latitude and longitude: 37.61666666, 101.200000. The voucher specimens were deposited at the Forestry Herbarium, College of Agriculture and Animal Husbandry, Qinghai University, Xining, China (wangchunjing00@163.com) under the voucher numbers: HB210330001.
Data accessibility	Raw data of *Ranunculus membranaceus* were deposited to NCBI Sequence Read Archive (SRA) under the BioProject PRJNA868870 (https://www.ncbi.nlm.nih.gov/bioproject/PRJNA868870) and BioSample SAMN30275514 (https://www.ncbi.nlm.nih.gov/biosample/30275514) with SRA number SRR21021544. Data identification number: PRJNA868870, SAMN30275514 The complete chloroplast genome of *Ranunculus membranaceus* is available in GenBank under accession number OL826870 (https://www.ncbi.nlm.nih.gov/nuccore/OL826870).

## Value of the Data

• *Ranunculus membranaceus* has significant medicinal value, and its complete chloroplast genome data will be useful in phylogenetic comparisons with the related species.
• The data will be useful to clarify the genus's chloroplast genome structure and in phylogenomic studies of *R. membranaceus*.
• The detected simple sequence repeats can be used in the development of potentially useful molecular markers and evaluation of genetic diversity in *R. membranaceus* populations and closely related species.

## 1 Objective

*Ranunculus membranaceus* Royle is an alpine perennial herb of the family Ranunculaceae, mainly distributed in the Qinghai-Tibet Plateau of China, northern India, Nepal, and Pakistan at altitudes between 2700 and 5000 m [1]. The whole plant, especially flowers, of *Ranunculus membranaceus* is commonly used in traditional Chinese medicine. The species is used to treat dyspepsia, laryngitis, abdominal lumps and hydrops, and arthritis. Its population declines due to grassland degradation and over-collection in the wild. Here, the complete chloroplast genome of *R. membranaceus* is described in order to provide a basis for resource management, conservation, breeding and investigating genetics of this medicinal species.

## 2 Data Description

**Fig. 1:**
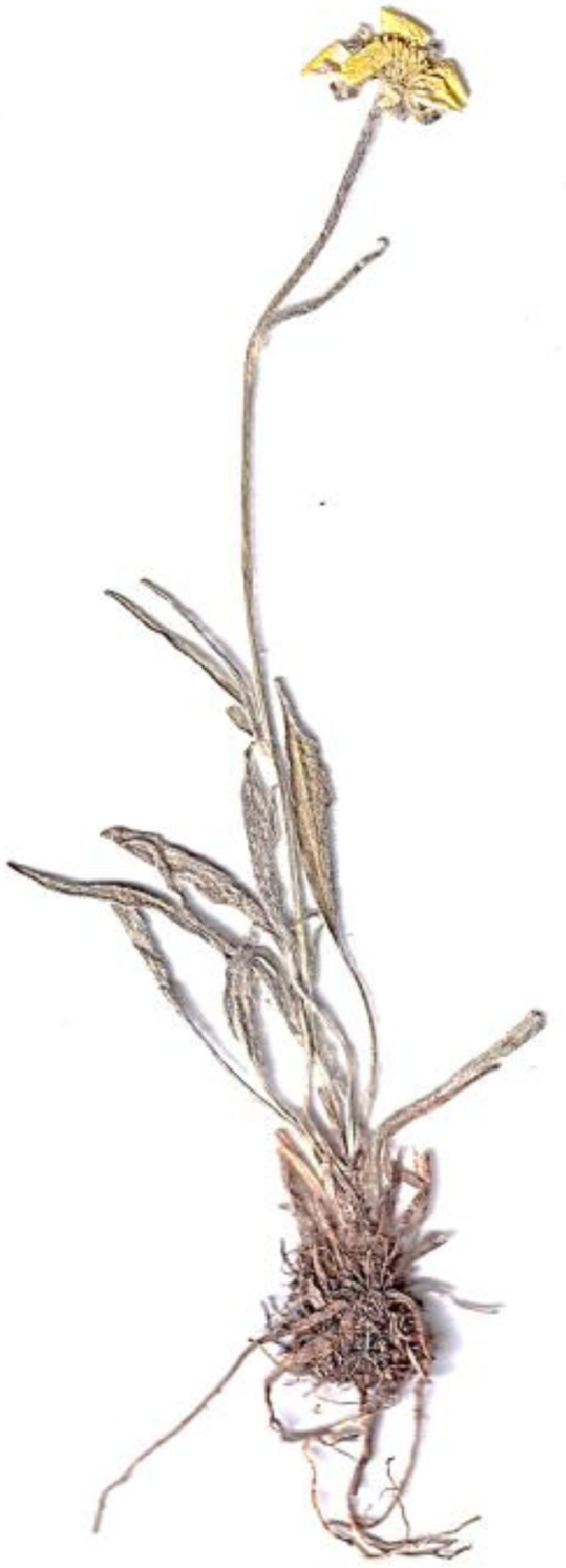
Image of *Ranunculus membranaceus*. This voucher specimen (the voucher number: HB210330001) was deposited at the Forestry Herbarium, College of Agriculture and Animal Husbandry, Qinghai University, Xining, China.

*R. membranaceus* is a species characterized by having fibrous roots, reduced sheathing basal leaves, deeply divided stem leaves, dense whitish-tomentose indument on all parts of the plant, and yellow flowers (Fig. 1) [1]. The whole plant, especially flowers, is commonly used in traditional Chinese medicine to warm the middle abdomen to dispel cold, invigorate the stomach and digestion, and clear damp to promote diuresis. It is used for dyspepsia, laryngitis, abdominal lumps and hydrops, and yellow water disease [2]. Here, the complete chloroplast genome of *R. membranaceus* is described, along with phylogenetic analysis of the cpDNA sequence information with 17 other species of the tribe Ranunculeae.

**Fig. 2:**
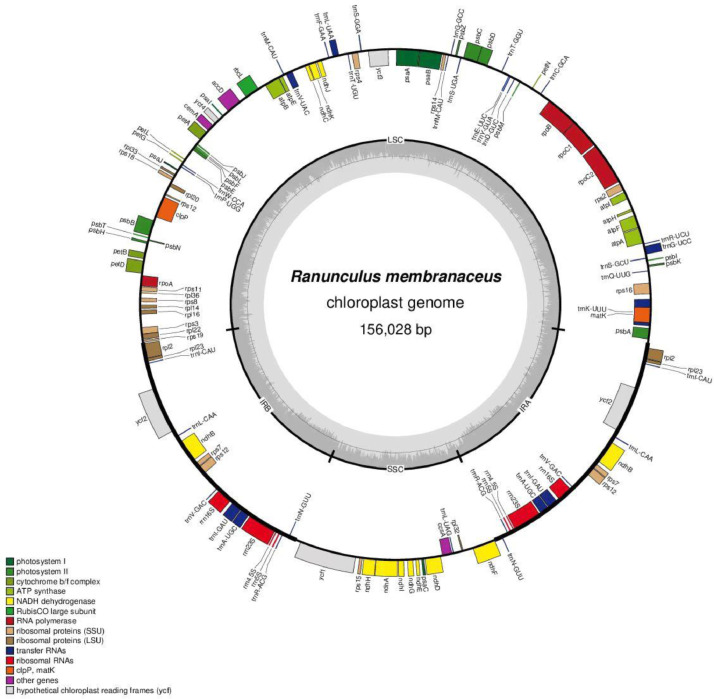
The chloroplast genome map of *Ranunculus membranaceus* in a circular diagram using OGDRAW. Genes shown outside the circle are transcribed clockwise, and genes inside are transcribed counter-clockwise. Genes belonging to different functional groups are color-coded. The darker grey in the inner circle corresponds to the GC content and the lighter grey to the AT content. The thick black line on the outer circle represents the two IR regions.

The *R. membranaceus* chloroplast genome (Fig. 2) was found to be 156,028 bp long. The mean coverage was 100.85x and the GC content was 37.9%. The assembled genome exhibited a tetrad structure with a large single-copy (LSC) region of 85,491 bp, two inverted repeats (IRs) of 25,361 bp, and a small single-copy (SSC) region of 19,815 bp. The GC contents of these regions differed, being highest in the IRs (43.5%), and lowest (36.1% and 31.5%) in the LSC and SSC regions, respectively.

**Fig. 3:**
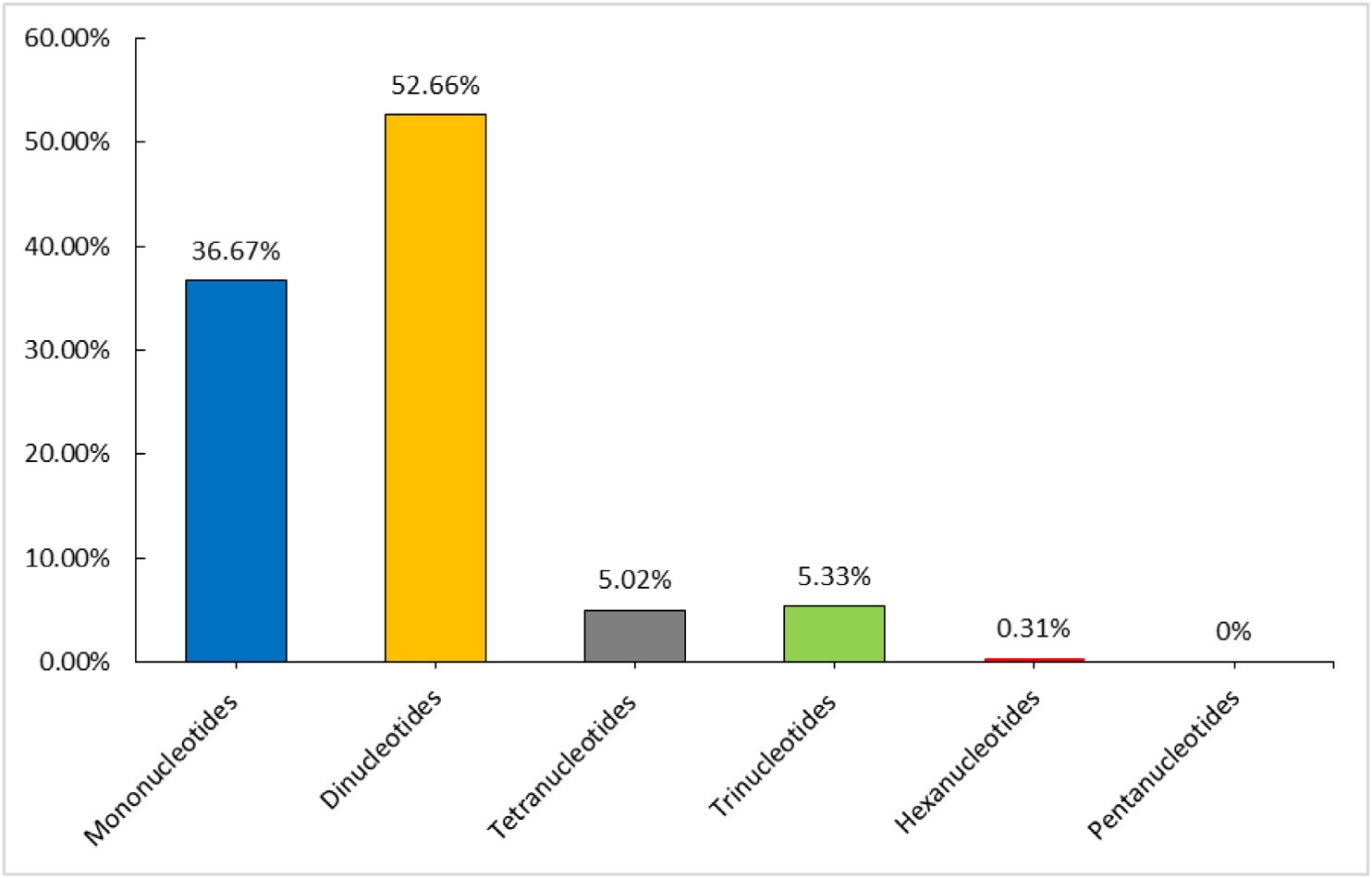
The percentage of each SSR type in *Ranunculus membranaceus*. The highest percentage of SSR motifs is represented by mononucleotides (36.7%), followed by dinucleotides (52.7%), tetranucleotides (5.0%), trinucleotides (5.3%), hexanucleotides (0.3%) and finally, pentanucleotides (0%).

One hundred and twenty-eight genes were observed (Table 1). Of these, 84 coded for protein, 36 for tRNA, and 8 for rRNA. Two copies of each of the tRNA genes *trnA-UGC, trnI-CAU, trnL-CAA, trnV-GAC, trnI-GAU*, and *trnR-ACG* were observed. Similarly, two copies of the rRNA genes *rrn4.5s* and *rrn5s* were identified. The *rpl2, rps7, rpl23*, and *ndhB* protein-coding genes also had two copies, while *rps12* had three copies. Three hundred and nineteen simple sequence repeats (SSRs) were found, of which dinucleotides were the most common SSR markers, representing 52.7% of the total. The remaining SSRs were composed of 117 (36.7%) mono-, 17 (5.3%) tetra-, 16 (5.0%) tri-, and 1 (0.3%) hexa-SSRs (Fig. 3).

**Table 1:** Classification of the *Ranunculus membranaceus* genes after annotation of the chloroplast genome. The annotated genes were categorized according to their function.

Groups of Genes	Genes
**Protein genes**	
ATP synthase	*atpA, atpI, atpB, atpF, atpH, atpE*
Cytochrome b/f complex	*petD, petN, petB, petL, petG, petA*
RubisCO large subunit	*rbcL*
NADH dehydrogenase	*ndhJ, ndhD, ndhK, ndhH, ndhA, ndhF, ndhI, ndhG, ndhC, ndhE, ndhB(× 2)*
Photosystem I	*psaJ, psaA, psaB, psaI, psaC*
Photosystem II	*psbF, psbM, psbB, psbC, psbA, psbH, psbN, psbL, psbZ, psbJ, psbT, psbD, psbK, psbE, psbI*
Hypothetical chloroplast reading frame	*ycf1, ycf4, ycf3, ycf2*
Ribosomal proteins	
Large subunits	*rpl14, rpl2(× 2), rpl16, rpl36, rpl22, rpl23(× 2), rpl32, rpl20, rpl33*
Small subunits	*rps19, rps15, rps14, rps11, rps7(× 2), rps18, rps4, rps12(× 3), rps2, rps16, rps8, rps3*
RNA polymerase	*rpoC2, rpoA, rpoC1, rpoB*
Acetyl-CoA carboxylase	*accD*
Inner envelope membrane	*cemA*
Cytochrome c biogenesis protein	*ccsA*
Protease	*clpP*
Maturase	*matK*
**RNAs Genes**	
ribosomal RNAs	*rrn23S, rrn16S, rrn4.5S(× 2), rrn5S(× 2)*
transfer RNAs	*trnG-UCC, trnY-GUA, trnfM-CAU, trnP-UGG, trnR-UCU, trnI-CAU(× 2), trnQ-UUG, trnV-GAC(× 2), trnT-UGU, trnS-GGA, trnV-UAC, trnF-GAA, trnS-GCU, trnL-UAG, trnN-GUU, trnW-CCA, trnI-GAU(× 2), trnK-UUU, trnG-GCC, trnT-GGU, trnM-CAU, trnL-CAA(× 2), trnC-GCA, trnE-UUC, trnR-ACG(× 2), trnS-UGA, trnD-GUC, trnL-UAA, trnA-UGC(× 2)*

**Fig. 4:**
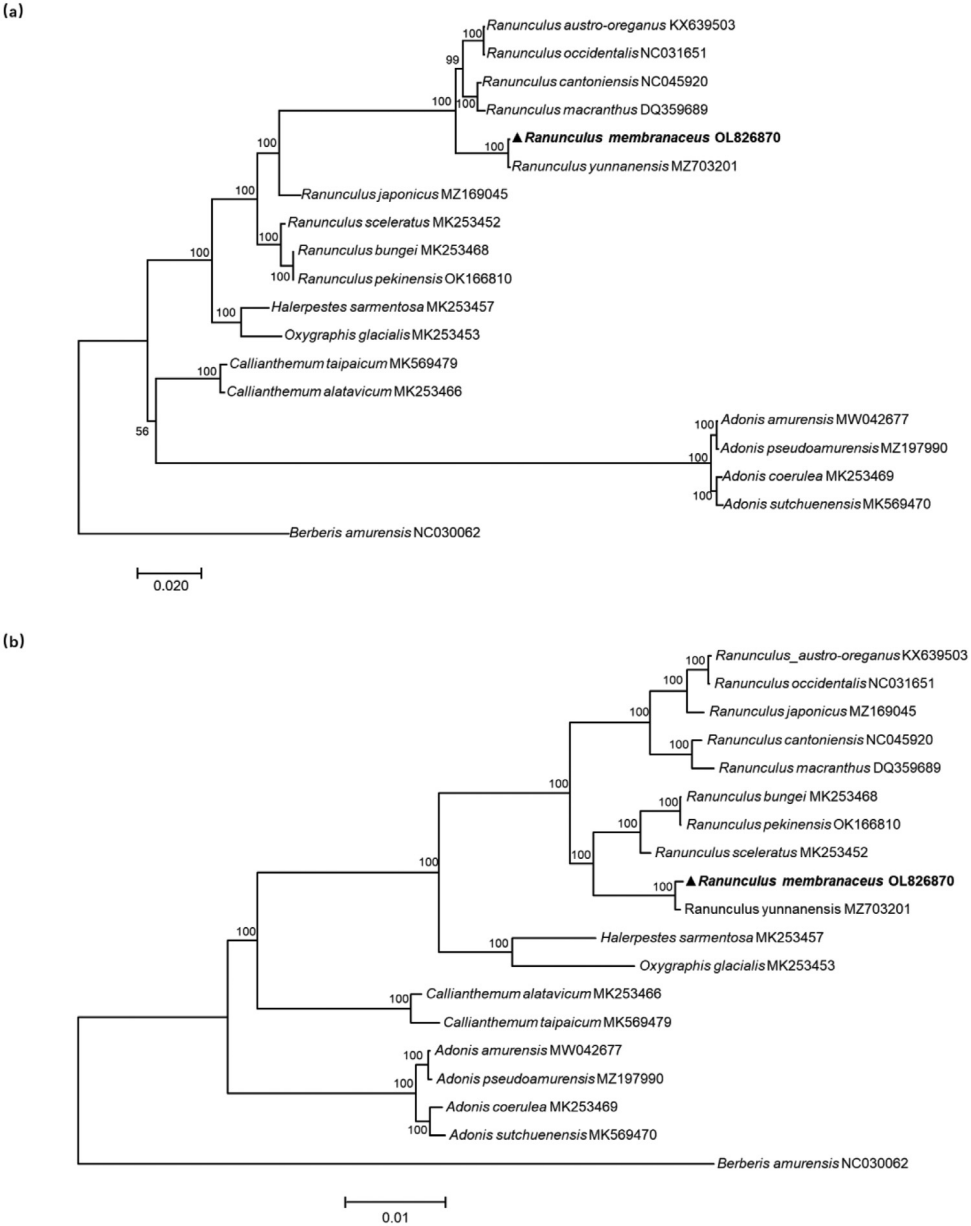
Maximum-likelihood phylogenetic trees the whole chloroplast genomes (a) and the concatenated sequence of PCGs (b) of 19 Ranunculaceae species. Numbers next to branches indicate bootstrap values (with 1000 replicates).

Two phylogenetic trees were constructed for *R. membranaceus* and 17 other species of the tribe Ranunculeae with *Meconopsis punicea* as the outgroup based on the whole chloroplast genomes and the concatenated sequence of PCGs, respectively. The phylogenetic tree based on the concatenated sequence of PCGs has a higher support rate than the phylogenetic tree based on the whole chloroplast genome. The topological structure of two phylogenetic trees is similar. It is apparent (Fig. 4) that *R. membranaceus* is closely related to *R. yunnanensis* with 100% bootstrap support in two phylogenetic trees. These findings enrich our knowledge of Ranunculaceae genetics, and provide a basis for resource management, breeding, and further investigations into the genetics of *R. membranaceus*.

## 3 Experimental Design, Materials and Methods

Fresh samples were collected from the Qinghai Haibei National Field Research Station of Alpine Grassland Ecosystem (altitude 3200 m, 37.617°N, 101.200°E), which is situated at Menyuan, Qinghai Province, China, in the north-eastern Qinghai-Tibet plateau [3]. A voucher specimen was deposited at the Forestry Herbarium, College of Agriculture and Animal Husbandry, Qinghai University, Xining, China under the voucher number HB210330001.

The chloroplast genome was sequenced using two sequencing methods, using the Illumina high-throughput sequencing platform Illumina NovaSeq 6000 with a 150-bp shotgun library and the Oxford Nanopore PromethION sequencer for real-time single-molecule sequencing. Illumina sequencing results were assembled via SPAdes (version 3.14.0) [4], and Nanopore sequencing results were assembled via Flye 2.8.3(https://github.com/fenderglass/Flye) [5]. The splicing results of Flye were compared using BLAST version 2.2.30^+^[6] with the chloroplast genome of *R. cantoniensis* as the reference [7]. GeSeq (online version: https://chlorobox.mpimp-golm.mpg.de/geseq.html) [8] was used for gene annotations with manual correction. Simple sequence repeats (SSRs) were analyzed using MISA (http://pgrc.ipk-gatersleben.de/misa/misa.html) with the following thresholds: eight for mononucleotide repeats, four for dinucleotide repeats, four for trinucleotide repeats, three for tetranucleotide repeats, three for pentanucleotide repeats, and three for hexanucleotide repeats. The annotated genome is available in GenBank under the accession number OL826870. The circular chloroplast genome map was drawn using OGDRAW [9].

The chloroplast genome data of 17 other species of the tribe Ranunculeae from NCBI were downloaded for the phylogenetic tree reconstruction. These species account for 61.2% of the total of species reported in the genus Ranunculus, and account for 20.2% in the tribe Ranunculeae. A phylogenetic tree of tribe Ranunculeae species was constructed using the maximum likelihood method with *Berberis amurensis* (GenBank: NC030062) as the outgroup. MAFFT [10,11] (online version: https://mafft.cbrc.jp/alignment/server/) was used to align the sequences using default settings and the tree was created using MEGA7 with the general time reversible (GTR) model with 1000 bootstraps [12]. After aligned, it was found that, the percentage of missing data is 39.5 %, the number of parsimony informative sites is 24568 (see the alignment in Appendix 1). For comparison with the ML phylogenetic tree based on complete chloroplast genomes, a ML phylogenetic tree based on the concatenated PCGs of 19 Ranunculaceae species was created using IQ-tree v1.6.8 [13] with 1000 bootstraps in PhyloSuite v1.2.2 [14]. Alignments were trimmed with PhyloSuite v1.2.2 [14] and manually edited where necessary. We used PartitionFinder2 in PhyloSuite v1.2.2 for optimal partitioning strategy and evolutionary model selection (see the best selected models in Appendix 2). Phylograms were visualized with FigTree v1.4.4 (http://tree.bio.ed.ac.uk/).

## Ethics Statements

This study did not require an official ethics review.

## CRediT Author Statement

All authors contributed to the design of the study and the writing of the manuscript. Material preparation and collection were performed by Chunhui Zhang, Lin Wu and Zhen Ma. The data analysis was performed by Yanmei Ren, Ji-zhong Wan, Mengyan Wang and Zuoyi Wang. The draft of the manuscript was written by Yanmei Ren. Huakun Zhou and Ji-Zhong Wan revisited critically for intellectual content and Chunhui Zhang and Zhen Ma reviewed the final version of our manuscript.

## Declaration of Competing Interest

The authors declare that they have no known competing financial interests or personal relationships that could have appeared to influence the work reported in this paper.

## Data Availability

Characterization of the complete chloroplast genome of Ranunculus membranaceus (Ranunculaceae) (Original data) (NCBI).

## Appendix Supplementary materials

Image, application 1

Image, application 2
